# Global Dynamics of a Virus Dynamical Model with Cell-to-Cell Transmission and Cure Rate

**DOI:** 10.1155/2015/758362

**Published:** 2015-10-01

**Authors:** Tongqian Zhang, Xinzhu Meng, Tonghua Zhang

**Affiliations:** ^1^College of Mathematics and Systems Science, Shandong University of Science and Technology, Qingdao 266590, China; ^2^State Key Laboratory of Mining Disaster Prevention and Control Co-Founded by Shandong Province and the Ministry of Science and Technology, Shandong University of Science and Technology, Qingdao 266590, China; ^3^Department of Mathematics, Swinburne University of Technology, P.O. Box 218, Hawthorn, VIC 3122, Australia

## Abstract

The cure effect of a virus model with both cell-to-cell transmission and cell-to-virus transmission is studied. By the method of next generation matrix, the basic reproduction number is obtained. The locally asymptotic stability of the virus-free equilibrium and the endemic equilibrium is considered by investigating the characteristic equation of the model. The globally asymptotic stability of the virus-free equilibrium is proved by constructing suitable Lyapunov function, and the sufficient condition for the globally asymptotic stability of the endemic equilibrium is obtained by constructing suitable Lyapunov function and using LaSalle invariance principal.

## 1. Introduction and Model Formulation

Since tobacco mosaic virus, the first virus of the world discovered by Beijerinck in 1898 [1], more and more viruses have been discovered by biologists, biomedical scientists, and medical scientists and more than 5,000 viruses have been recorded in detail [2]. However, according to a recent study, there are at least 32,0000 viruses waiting to be discovered in the spread between mammalian species. Identifying diseases caused by these viruses, especially those that can infect people, perhaps can help us to prevent epidemic disease [3]. At the early stage of the study, it is generally accepted that because of the specificity of viruses, virus can only infect certain plant or animal species; however, more and more cases associated with emerging zoonoses have appeared, and with a deeper understanding of the virus, we found that most viruses can infect humans, such as Human Immunodeficiency Virus (HIV), Prions, Influenza Virus, Rabies Virus, Ebola Virus, and Middle East Respiratory Syndrome Coronavirus (MERSV) [3–6].

Generally, the basic process of viral infection and virus replication occurs in six main steps: attachment, penetration, uncoating, replication, assembly, and release [7]. After the whole replicative cycle, free viruses begin to diffuse and infect new host cell. Therefore, investigating the processes of viral growth and destruction of host cells so as to gain the insights into the evolutionary processes of virus and cell in body is very important. To this end, mathematical models and analysis are powerful tools.

Since mathematical models and method of mathematical analysis were used to study the dynamics of the virus, lots of models have been established to explain the evolution of the uninfected target cells, infected cells, and the free virus. In these models, early works belonged to Nowak et al. [8], Nowak and May [9], Perelson and Nelson [10], and Perelson et al. [11]. The general class of models that have been studied [8–12] have a form similar to

(1) dxdt=Λ−dx−αvx,dydt=αvx−ay,dvdt=ky−uv,

where *x*, *y*, and *v* represent the concentrations of uninfected target cells, infected cells, and virus, respectively. For explanations of other parameters we refer to literature [12]. This model describes the processes of virus invading the target cells and the release of the virus due to the infected cell apoptosis. In the model, the authors use *αvx* to represent the interaction between uninfected target *x* and virus *v*, which obey the principle of mass action. Based on model (1), more authors used nonlinear functions to describe the rate constant characterizing infection of cells, for example, *βxv*/(*x* + *y*) in [13], *βxv*/(1 + *bv*) in [14, 15], *βxv*/(1 + *ax* + *bv*) in [16], and *βxv*/(1 + *ax* + *bv* + *abxv*) in [17], and for details of more general nonlinear incidence rate functions please see [18–20]. Notice that there exists a potentially possible cure rate of the infected cells to the susceptible host cells in the infection process of some virus, such as Hepatitis B Virus (HBV) [21–25] and HIV [26–32]; recently, Hattaf et al. [19] adopted a general nonlinear incidence rate function with the form *f*(*x*, *y*, *v*)*v* and introduced cure rate (denoted by *ρ*) into the following model:

(2) dxdt=Λ−dx−fx,y,vv+ρy,dydt=fx,y,vv−a+ρy,dvdt=ky−uv.

In model (2), *f*(*x*, *y*, *v*) satisfies the following hypotheses:

$\left(H_{1}^{\prime }\right)$ *f*(0, *y*, *v*) = 0, for all *y* ≥ 0.
$\left(H_{2}^{\prime }\right)$ ∂*f*(*x*, *y*, *v*)/∂*x* > 0, for all *x* > 0, *y* ≥ 0, and *v* ≥ 0.
$\left(H_{3}^{\prime }\right)$ ∂*f*(*x*, *y*, *v*)/∂*y* ≤ 0 and ∂*f*(*x*, *y*, *v*)/∂*v* ≥ 0, for all *x* ≥ 0, *y* ≥ 0, and *v* ≥ 0.

Recently, Tian and Liu [20] improved model (2) by proposing a more general nonlinear incidence rate function with the form *f*(*x*, *y*, *v*) and investigated the following model:

(3) dxdt=Λ−dx−fx,y,v+ρy,dydt=fx,y,v−a+ρy,dvdt=ky−uv.

In model (3), *f*(*x*, *y*, *v*) satisfies the following hypotheses:

$\left(H_{1}\right)$ *f*(0, *y*, *v*) = 0, for all *y* ≥ 0 and *v* ≥ 0, and *f*(*x*, *y*, 0) = 0, for all *x* ≥ 0 and *y* ≥ 0.
$\left(H_{2}\right)$ ∂*f*(*x*, *y*, *v*)/∂*x* > 0, for all *x* ≥ 0, *y* ≥ 0, and *v* > 0.
$\left(H_{3}\right)$ ∂*f*(*x*, *y*, *v*)/∂*y* ≤ 0, for all *x* ≥ 0, *y* ≥ 0, and *v* ≥ 0.
$\left(H_{4}\right)$ ∂*f*(*x*, *y*, *v*)/∂*v* ≥ 0 and *v*(∂*f*(*x*, *y*, *v*)/∂*v*) − *f*(*x*, *y*, *v*) ≤ 0, for all *x* ≥ 0, *y* ≥ 0, and *v* ≥ 0.

However, many researches show that direct cell-to-cell spread can happen in some enveloped viruses (e.g., Human Immunodeficiency Virus type-1 (HIV-1) [33–37], Human T-Lymphotropic Virus Type-1 (HTLV-1) [38–41], Herpes Simplex Virus (HSV) [42], and Measles [43–45]). Cell-to-cell spread not only facilitates rapid viral dissemination, but may also promote immune evasion and influence disease [46]. Moreover, a recent study has shown that cell-to-cell spread of HIV-1 can reduce the sensitivity to the antiretroviral drugs by multiple infections of target cells and, as a result, the efficacy of antiretroviral therapy is reduced [47].

Motivated by the works [18–20, 48], we propose a virus dynamical model with both cell-to-virus infection and cell-to-cell transmission and cure rate as follows:

(4) dxdt=Λ−dx−fy,vx+ρy,dydt=fy,vx−a+ρy,dvdt=ky−uv,

where *x*, *y*, and *v* denote the number of host cells, infected cells, and free virus, respectively. And *d*, *a*, and *u* are the death rates of them, respectively. Free virus is produced by infected cells at a rate *ky*. Λ represents the regeneration rate of host cells. *ρ* is the cure rate. *f*(*y*, *v*)*x* = (*βy* + *αv*)*x* represents the total infection rate of host cells, which is divided into two parts *βyx* and *αvx*. The former represents the part where infected cells infect host cells by direct contact, and the latter means that host cells are infected by the free virus. For more detail, please see [48]. In the present model, we can see *f*(*y*, 0)*x* = *βxy*≢0, for all *x* ≥ 0 and *y* ≥ 0, and ∂*f*(*y*, *v*)*x*/∂*y* = *βx* ≥ 0 for all *x* ≥ 0, *y* ≥ 0, and *v* ≥ 0, which do not satisfy conditions (*H*_3_′) in model (2) and conditions (*H*_1_) and (*H*_3_) in model (3). For biological considerations, we will study system (4) in the closed set *A* = {(*x*, *y*, *v*) ∈ *R*_+_^3^∣*x* + *y* ≤ Λ/*d*, *v* ≥ 0}.

The main goal of the present paper is to investigate the globally asymptotic stability of the equilibria of (4). This work is structured as follows. In Section 1, we give the motivation and study the background of the model. In Section 2, the existence of virus-free equilibrium and the endemic equilibrium is shown based on the basic reproduction number. And the local stability of the two equilibria is discussed in Section 3. We focus on the globally asymptotic stability of the two equilibria in Section 4. Finally, a brief conclusion and discussion are given in Section 5.

## 2. Basic Reproduction Number and Equilibria

The basic reproduction number [49, 50] of model (4) is given as

(5) $$\begin{array}{c} R=\frac{\Lambda \left(\alpha k+\beta u\right)}{du\left(a+\rho \right)}. \end{array}$$

Based on the basic reproduction number *ℛ*, we have Theorem 1.

**Theorem 1 .**

Model (4) always has a virus-free equilibrium *E*_0_ = (*x*_0_, 0,0), where *x*_0_ = Λ/*d*. If *ℛ* > 1, model (4) has a unique endemic equilibrium *E*_1_(*x* ^*∗*^, *y* ^*∗*^, *v* ^*∗*^), where

(6) x∗=ΛdR,y∗=Λa1−1R,v∗=kuy∗.

## 3. Local Stability of the Two Equilibria

In this section, we shall show the local stability of equilibria *E*_0_ and *E*_1_.

**Theorem 2 .**

For model (4), we have the following conclusion:

(i) *E*_0_ is locally stable if *ℛ* < 1 and unstable if *ℛ* > 1.
(ii) *E*_1_ is locally stable if *ℛ* > 1.

**Proof**

We firstly prove (i). Notice the Jacobian of model (4) evaluated *E*_0_ is given by

(7) $$\begin{array}{c} J\left(\;E_{0}\;\right)=\left(\begin{array}{ccc} -d & \rho -\beta x_{0} & -\alpha x_{0}\\ 0 & \beta x_{0}-\left(a+\rho \right) & \alpha x_{0}\\ 0 & k & -u \end{array}\right). \end{array}$$

Obviously, *J*(*E*_0_) has an eigenvalue *λ* = −*μ* < 0, and the other two eigenvalues *λ*_2_ and *λ*_3_ satisfy

(8) λ2+λ3−a+ρ−βx0+u=−αkuR+βx01R−1+u,λ2λ3a+ρ−βx0u−αkx0=a+ρu1−R.

Then, when *ℛ* < 1, *λ*_2_ + *λ*_3_ < 0, and *λ*_2_ *λ*_3_ > 0, all the eigenvalues of *J*(*E*_0_) have negative real parts and *E*_0_ is locally asymptotically stable. And when *ℛ* > 1 and *λ*_2_ *λ*_3_ < 0, *J*(*E*_0_) has a positive eigenvalue and *E*_0_ is unstable.

Next, we prove (ii). The Jacobian of model (4) evaluated *E*_1_ is

(9) $$\begin{array}{c} J\left(\;E_{1}\;\right)=\left(\begin{array}{ccc} -d-\alpha v^{*}-\beta y^{*} & \rho -\beta x^{*} & -\alpha x^{*}\\ \alpha v^{*}+\beta y^{*} & \beta x^{*}-\left(a+\rho \right) & \alpha x^{*}\\ 0 & k & -u \end{array}\right), \end{array}$$

from which we have the characteristic equation

(10) $$\begin{array}{c} A\lambda^{3}+B\lambda^{2}+C\lambda +D=0, \end{array}$$

where

(11) A=ux∗,B=ρy∗u+Λu+x∗u2+x∗2αk,C=Λαx∗k+Λu2+ρy∗αx∗k+ρy∗u2−y∗uaρ−y∗uρ2+y∗uaβx∗+y∗uρβx∗,D=y∗uaαx∗k+y∗u2aβx∗+y∗u2ρβx∗−y∗u2aρ−y∗u2ρ2+y∗uραx∗k.

Obviously, *A*, *B* > 0. And noticing that *x* ^*∗*^(*uβ* + *αk*) = *u*(*a* + *ρ*), we have

(12) C=Λαx∗k+Λu2+ρy∗αx∗k+ρy∗u2−y∗uaρ−y∗uρ2+y∗uaβx∗+y∗uρβx∗=Λαx∗k+Λu2+ρy∗u2+y∗uaβx∗+ρy∗x∗αk+uβ−ρy∗ua+ρ=Λαx∗k+Λu2+ρy∗u2+y∗uaβx∗>0,D=y∗uaαx∗k+y∗u2aβx∗+y∗u2ρβx∗−y∗u2aρ−y∗u2ρ2+y∗uραx∗k=y∗uaαx∗k+y∗u2aβx∗+ρuy∗x∗uβ+αk−ρy∗u2a+ρ=y∗uaαx∗k+y∗u2aβx∗>0,BC−AD=ρy∗u+Λu+x∗u2+x∗2αk·Λαx∗k+Λu2+ρy∗u2+y∗uaβx∗−ux∗y∗uaαx∗k+y∗u2aβx∗=ρy∗u+Λu+x∗2αk+x∗u2·Λαx∗k+y∗uaβx∗+Λu2+ρy∗u2−ux∗y∗uaαx∗k+y∗u2aβx∗=ρy∗u+Λu+x∗2αk·Λαx∗k+y∗uaβx∗+Λu2+ρy∗u2+x∗u2Λu2+ρy∗u2+x∗u2Λαx∗k+y∗uaβx∗−ux∗y∗uaαx∗k+y∗u2aβx∗=ρy∗u+Λu+x∗2αk·Λαx∗k+y∗uaβx∗+Λu2+ρy∗u2+x∗u2Λu2+ρy∗u2+x∗u2Λαx∗k−ux∗y∗uaαx∗k=ρy∗u+Λu+x∗2αk·Λαx∗k+y∗uaβx∗+Λu2+ρy∗u2+x∗u2Λu2+ρy∗u2+x∗2ku2αΛ−y∗a>0,

where *ℛ* > 1 and *y* ^*∗*^ = Λ/*a*(1 − 1/*ℛ*) < Λ/*a* are used. Then, by the Routh-Hurwitz Criterion [51], we know that all the roots of (10) always have negative real parts. Thus, the epidemic equilibrium *E*_1_ is locally asymptotically stable for *ℛ* > 1.

## 4. Global Stability of the Two Equilibria

In this section, we study the global behaviors of model (4) by constructing Lyapunov functions. Firstly, we show the global stability of *E*_0_.

**Theorem 3 .**

If *ℛ* < 1, the virus-free equilibrium *E*_0_ is globally asymptotically stable.

**Proof**

Define a Lyapunov function *L*_1_(*t*) on *A* as follows:

(13) L1=x−x0−x0ln⁡xx0+ρ2d+ax0x−x0+y2+y+pv;

here, *p* > 0 is a constant to be determined. It follows from (4) and (13) that

(14) dL1dt=Λ−dx−βy+αvx+ρy−x0xΛ−dx−βy+αvx+ρy+ρ2d+ax02x−x0+y·Λ−dx−βy+αvx+ρy+βy+αvx−a+ρy+βy+αvx−a+ρy+pky−uv=dx0−x−dx0xx0−x+ρy+βy+αvx0−x0xρy+ρd+ax0x−x0+y·dx0−x−ay−a+ρy+pky−uv=−dxx0−x2+βy+αvx0+ρy−x0x·ρy+ρd+ax0−dx−x02+a+dyx0−x−ay2−a+ρy+pky−uv=−dxx0−x2+βy+αvx0−ρyxx0x0−x2−dρd+ax0x−x02−aρd+ax0y2−a+ρy+pky−uv=−dxx0−x2−ρyxx0x0−x2−dρd+ax0x−x02−aρd+ax0y2+kp−a+ρ−βx0ky+uαx0u−pv.

Since *ℛ* < 1, we have (*βk* + *αu*)*x*_0_ < *u*(*a* + *ρ*); then, we can choose *p* > 0 such that *βx*_0_/*u* < *p* < (*a* + *ρ* − *αx*_0_)/*k*. Hence, we have that *dL*_1_(*t*)/*dt* < 0. Then, *E*_0_ is globally asymptotically stable.

Next, we study the global stability of the endemic equilibrium *E*_1_.

**Theorem 4 .**

If 1 < *ℛ* ≤ 1 + *δ*, the epidemic equilibrium *E*_1_ is globally asymptotically stable, where $\delta =\left(\beta \Lambda +(a-\rho )d+\sqrt{{\left(\beta \Lambda +(a-\rho )d\right)}^{2}+4a\rho d^{2}}\right)/2\rho d.$

**Proof**

If *ℛ* > 1, we define a Lyapunov function *L*_2_(*t*) as follows:

(15) L2t=x−x∗−x∗ln⁡xx∗+y−y∗−y∗ln⁡yy∗+αx∗v∗ky∗v−v∗−v∗ln⁡vv∗+ρ2d+ax−x∗+y−y∗2.

It follows from (4) and (15) that

(16) dL2tdt=Λ−dx−βy+αvx+ρy−x∗xΛ−dx−βy+αvx+ρy+βy+αvx−a+ρy−y∗yβy+αvx−a+ρy+αx∗v∗ky∗ky−uv−v∗vky−uv+ρ2d+ax∗2x−x∗+y−y∗·Λ−dx+ρy−a+ρy=−dx−x∗2x+βx∗y∗+αx∗v∗−ρy∗+ρy−x∗xβx∗y∗−x∗x·αx∗v∗+x∗xρy∗+x∗βy+x∗αv−x∗xρy−a+ρy−y∗βx−y∗yαvx+βx∗y∗+αx∗v∗+αx∗v∗ky∗ky−uv−v∗vky−uv−dρd+ax∗x−x∗2−aρd+ax∗y−y∗2+ρx∗x−x∗y∗−y=−dx−x∗2x−βy∗·x−x∗2x+αx∗v∗2−x∗x+vv∗−y∗vxx∗v∗y−yy∗+αx∗v∗yy∗−vv∗−v∗yvy∗+1+ρxx−x∗·y−y∗−dρd+ax∗x−x∗2−aρd+ax∗y−y∗2+ρx∗x−x∗y∗−y=−dx−x∗2x−βy∗x−x∗2x+αx∗v∗3−x∗x−y∗vxx∗v∗y−v∗yvy∗−dρd+ax∗x−x∗2−aρd+ax∗y−y∗2−ρxx∗x−x∗2y−y∗=−dx∗+dρxd+a+ρy−y∗+βx∗y∗·x−x∗2xx∗+αx∗v∗3−x∗x−y∗vxx∗v∗y−v∗yvy∗−aρd+ax∗y−y∗2=−dx∗+βx∗y∗−ρy∗+dρxd+a+ρy·x−x∗2xx∗+αx∗v∗3−x∗x−y∗vxx∗v∗y−v∗yvy∗−aρd+ax∗y−y∗2.

Since the arithmetic mean is greater than or equal to the geometric mean, it follows that

(17) $$\begin{array}{c} 3-\frac{x^{*}}{x}-\frac{y^{*}vx}{x^{*}v^{*}y}-\frac{v^{*}y}{vy^{*}}\leq 0. \end{array}$$

The above equality holds only for *x* = *x* ^*∗*^, *y* = *y* ^*∗*^, and *v* = *v* ^*∗*^. Clearly, if *ℛ* > 1 and *dx* ^*∗*^ − *βx* ^*∗*^ *y* ^*∗*^ − *ρy* ^*∗*^ > 0, then *dL*_2_(*t*)/*dt* ≤ 0. Note that *dx* ^*∗*^ − *βx* ^*∗*^ *y* ^*∗*^ − *ρy* ^*∗*^ ≥ 0 can be formulated as

(18) 1<R≤1+βΛ+a−ρd+βΛ+a−ρd2+4aρd22ρd=1+δ.

Since *dL*_2_(*t*)/*dt* = 0 if and only if *x* = *x* ^*∗*^, *y* = *y* ^*∗*^, and *v* = *v* ^*∗*^, by LaSalle invariance principle [52], the equilibrium *E*_1_ is globally asymptotically stable.

## 5. Conclusion and Discussion

**Figure 1:**
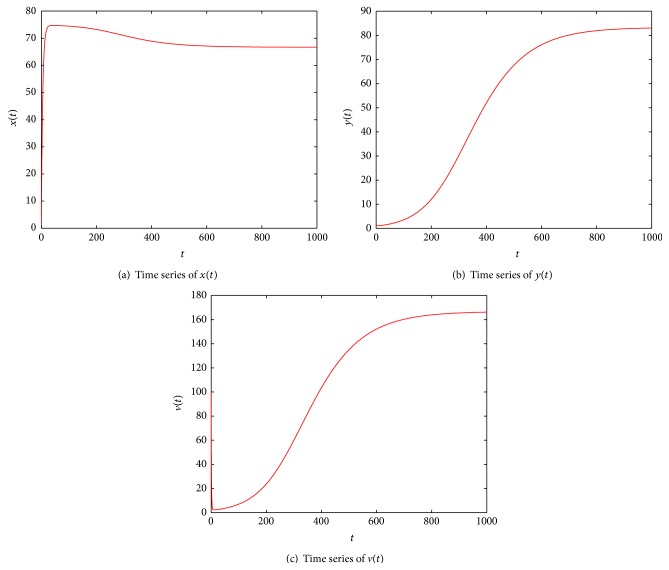
Illustration of numerical solution of system (4) with *λ* = 15, *d* = 0.2, *β* = 0.0008, *α* = 0.0005, *ρ* = 0.1, *a* = 0.02, *k* = 2, and *u* = 1, and *x*(0) = 1, *y*(0) = 1, and *v*(0) = 100. By calculation, one gets that *ℛ* = 1.125 and *δ* = 0.3583; it is easy to verify 1 < *ℛ* = 1.125 < 1 + *δ* = 1.3583; then, the equilibrium *E*_1_ is globally asymptotically stable.

**Figure 2:**
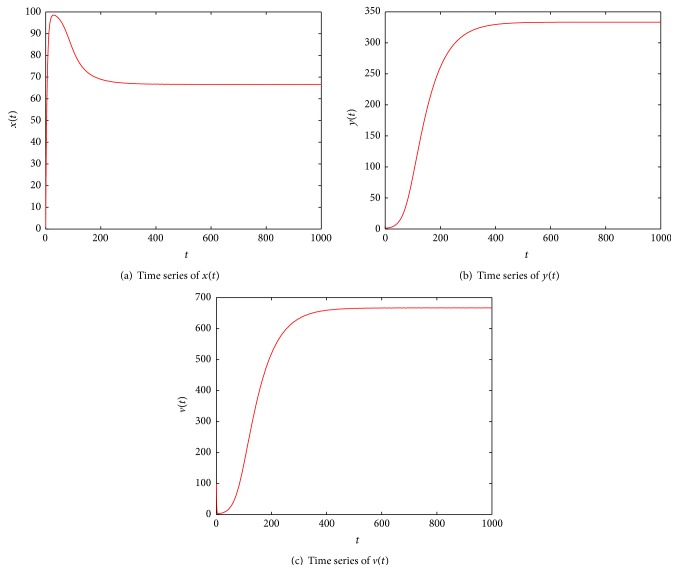
Illustration of numerical solution of system (4) with *λ* = 20, *d* = 0.2, *β* = 0.0008, *α* = 0.0005, *ρ* = 0.1, *a* = 0.02, *k* = 2, and *u* = 1, and *x*(0) = 1, *y*(0) = 1, and *v*(0) = 100. By calculation, one gets that *ℛ* = 1.5 and *δ* = 0.4472; it is easy to verify 1 < *ℛ* = 1.5 > 1 + *δ* = 1.4472, while the equilibrium *E*_1_ is also globally asymptotically stable.

In this paper, we considered the cure effect of a virus model with both cell-to-cell transmission and cell-to-virus transmission. By the method of next generation matrix, the basic reproduction number *ℛ* is obtained. Firstly the locally asymptotic stability of the virus-free equilibrium and the endemic equilibrium is considered. Then, the globally asymptotic stability of the virus-free equilibrium is proved by constructing suitable Lyapunov function, and the sufficient condition for the globally asymptotic stability of the endemic equilibrium is obtained by constructing suitable Lyapunov function and using LaSalle invariance principal. By analyzing the condition for the globally asymptotic stability of the endemic equilibrium, we have that if *ρ* = 0, from Theorem 4, the conditions *ℛ* > 1 can ensure the global stability of the equilibrium *E*_1_, While if *ρ* > 0, by the numerical simulations (see Figures 1 and 2), we find that *ℛ* ≤ 1 + *δ* in Theorem 4 is not necessary and can be dropped.
